# The Effect of Solution-Focused Brief Group Counseling on Adolescents’ Self-Confidence Levels

**DOI:** 10.3390/bs16030378

**Published:** 2026-03-06

**Authors:** Okan Bilgin, Mustafa Koç

**Affiliations:** 1 Department of Educational Sciences, Faculty of Education, Zonguldak Bülent Ecevit University, 67100 Zonguldak, Türkiye; 2 Department of Educational Sciences, Faculty of Education, Düzce University, 81620 Düzce, Türkiye; mkoc@duzce.edu.tr

**Keywords:** solution-focused brief counseling, self-confidence, adolescence

## Abstract

Solution-focused brief group counseling is a systematic approach that centers on solutions rather than the problem itself. In this approach, which focuses on the strengths of the clients, the solutions are generated by the clients themselves, which is theorized to positively affect their self-confidence. The purpose of this study is to examine the effect of Solution-Focused Brief Counseling (SFBC) on adolescents’ self-confidence levels. In line with the purpose of the study, the following hypothesis was tested: SFBC will significantly increase the self-confidence levels of adolescents with low and moderate self-confidence, and these gains will be maintained during the follow-up period. Utilizing a 2 × 3 mixed design (experimental/control groups × pre-test/post-test/follow-up), participants with low and moderate self-confidence scores were randomly assigned to either experimental or control groups. The adolescents in the experimental groups received a six-session SFBC intervention developed by the researcher. Data were collected using the Self-Confidence Scale and a personal information form. According to the findings, SFBC provided a statistically significant increase in the self-confidence levels of the adolescents in the experimental groups for both low and moderate baseline levels. These improvements were successfully maintained during the follow-up period, whereas no significant differences were found across the pre-test, post-test, and follow-up measurements of the participants in the control groups.

## 1. Introduction

Adolescence is a period of preparation and transition from childhood to adulthood when a great number of physical, mental, cognitive, social, and moral developments take place (Suner, 2000). In adolescence, students have to deal with many problems resulting both from the society and education system they live in and the characteristics of developmental periods. Getting over these problems in a healthy and proper way is very important in terms of adolescents’ characters and academic developments (Sarıcı-Bulut, 2008). Lack of self-confidence is considered one of the most important problems experienced during adolescence (Bilgin, 2011).

### 1.1. Self-Confidence in Adolescence

Self-confidence is a subjective phenomenon that occurs as a result of an individual assessing themself and being pleased or unpleased with themself (Mckay & Fanning, 2009). It involves developing positive feelings for oneself, being at peace with oneself, and feeling sufficient (Eldeleklioğlu, 2004; Altıntaş, 2015). Self-confidence that has not been gained in childhood will negatively influence an individual’s life, especially during adolescence when the individual often experiences identity confusion (Bilgin, 2011). However, a crucial feature of self-confidence is that it can be learned and developed later in life (Göknar, 2010; Altıntaş, 2015).

To contextualize the importance of this construct, prior empirical findings can be grouped conceptually. Studies indicate that low self-confidence in adolescence is negatively associated with internalizing symptoms and behavioral problems, such as anxiety (Merey, 2010), stress and health complaints (Hildingh et al., 2006), abnormal eating behaviors (Alşan, 2005), and peer bullying (Umutlu, 2010). Conversely, self-confidence is positively associated with psychological resilience (Akkuş, 2015), personal initiative (Okyay, 2012), leadership levels (Kurtuldu, 2007), and academic achievement (Soner, 1995; Özcan, 1996). Since a low level of self-confidence negatively affects many aspects of an adolescent’s life, professional counseling support is essential to help adolescents overcome these issues.

### 1.2. Solution-Focused Brief Therapy (SFBT) and Its Mechanisms

The personal, social, and developmental needs of adolescents differ from those of adult client populations. Therefore, methods and approaches applied during the psychological counseling process should be tailored specifically for them (Lines, 2006). Adolescents often prefer psychological help processes that do not deeply analyze their problems, which they might perceive as criticism from the adult world, but rather focus on finding immediate and practical solutions (Macdonald, 2007).

The Solution-Focused Brief Counseling approach, developed by Steve de Shazer and Insoo Kim Berg, advances by focusing on the solution rather than the problem, using limited time (De Shazer et al., 1986; Gladding, 2013; Sklare, 2010). The central principles of this therapy are based on three basic rules: “if it isn’t broken, don’t fix it,” “once you know what works, do more of it,” and “if it doesn’t work, try something different” (Berg & Miller, 1992).

The link between SFBT mechanisms and self-confidence development is rooted in the approach’s core principles. SFBT provides a conceptual framework that directly supports developmental factors in adolescents by validating their autonomy. For instance, the “miracle question” technique helps adolescents envision a future unburdened by self-doubt, fostering hope and identifying hidden goals. “Scaling questions” allow them to recognize small, concrete increments of progress, which directly builds self-efficacy and internal motivation. Furthermore, by emphasizing strengths and exploring “exception questions” (times when a problem was absent or manageable), SFBT reframes the adolescent’s self-concept from one of failure to one of competence. The solution-focused approach creates a positive effect on the client’s self-confidence by not focusing on things that a person did not try or could not do so far, but on their previous successes.

A growing body of literature highlights the positive outcomes of solution-focused interventions across various psychological domains. When prior findings are grouped conceptually, studies have demonstrated its benefits in reducing internalizing and externalizing symptoms, such as depression, anxiety, burnout, risk-taking behavior, and peer bullying (Gingerich & Wabeke, 2001; Eisengart & Gingerich, 2000; İlbay, 2014; Uysal, 2014; Çitemel, 2014). More importantly, it has been positively associated with self-related constructs, including self-esteem and self-efficacy (Springer et al., 2000; Kvarme et al., 2010; Bingöl, 2015).

### 1.3. The Present Study

Experts who adopt a solution-focused approach believe that providing clues to the solution and discussing exceptional moments increase individuals’ self-confidence (Murphy, 2008). Despite this strong theoretical relationship, there is a gap in the literature regarding empirical studies directly examining the effect of the solution-focused brief approach specifically on self-confidence in adolescence.

Thus, the purpose of this study is to examine the effect of Solution-Focused Brief Counseling on increasing the self-confidence levels of adolescents. To expand the scope of the study, it examined how the intervention affected adolescents with moderate levels of self-confidence in addition to those with low levels.

In line with the purpose of the study, the primary hypothesis is:

- Solution-Focused Brief Counseling will significantly increase the level of self-confidence of adolescents.

The secondary (sub)hypothesis is:

- There will be a significant difference between the self-confidence levels of the experimental groups (who received the intervention) and the control groups for both low and moderate self-confidence levels, and these improvements will be maintained during the follow-up period.

## 2. Materials and Methods

### 2.1. Study Design

This study utilized a randomized pre-test–post-test control group experimental design. The dependent variable is the participants’ self-confidence levels (measured by the Self-Confidence Scale), and the independent variable is the 6-session Solution-Focused Brief Group Counseling intervention. To test the hypotheses, a 2 × 3 mixed design was employed, where the first factor represents the experimental conditions (intervention vs. no-intervention control) and the second factor represents the repeated measurements (pre-test, post-test, and follow-up test). Utilizing a control group helped mitigate the effects of confounding variables, strengthening the causal inferences of the intervention. The overall study design is summarized in Table 1.

### 2.2. Data Collection Tools

#### Self-Confidence Scale

To address the measurement of the dependent variable, the Self-Confidence Scale developed by Akın (2007) was utilized. The scale consists of 33 items structured on a 5-point Likert-type scale ranging from 1 (“Never”) to 5 (“Always”). Higher total scores indicate higher levels of self-confidence. Based on the scale’s evaluation criteria, average item scores are interpreted as follows: a score below 2.5 indicates a low self-confidence level, a score between 2.5 and 3.5 indicates a moderate self-confidence level, and a score of 3.5 and above indicates a high self-confidence level.

During its development, factor analysis revealed a two-factor structure (inner and outer self-confidence) explaining 43.6% of the total variance. Criterion validity was established by correlating it with the Coopersmith Self-Esteem Inventory, yielding a high correlation (r = 0.87). For reliability, the original internal consistency coefficient was 0.83, and the test–retest reliability was 0.94. In the current study, the Cronbach’s alpha internal consistency coefficient for the whole scale was found to be highly reliable at 0.91.

### 2.3. Participants and Group Formation

The participants initially involved 558 students (ages 14–18) studying in the 9th, 10th, and 11th grades of a high school in Turkey. First, the Self-Confidence Scale was administered to this large pool to identify their baseline self-confidence levels. Using the scale’s cut-off scores (average <2.5 for low; 2.5–3.5 for moderate), students with low and moderate self-confidence were identified.

After informing these students about the study, volunteers were selected. A simple randomization procedure (drawing names blindly from a hat) was used to ensure every volunteering student had an equal probability of being assigned to a specific condition. This process resulted in 48 adolescents being assigned to four separate groups (n = 12 per group):

1. Low self-confidence experimental group.
2. Low self-confidence control group.
3. Moderate self-confidence experimental group.
4. Moderate self-confidence control group.

The experimental groups received the intervention, while the control groups served as a no-intervention baseline and did not receive any counseling or alternative activities during this period. The final sample consisted of 21 females and 27 males across the four groups. The distribution of the participants in terms of gender and grade across the experimental and control groups is presented in Table 2.

### 2.4. Development Process of the Intervention

Prior to developing the intervention, the researcher completed formal training in solution-focused brief therapy. Drawing upon the core principles of de Shazer and Berg, a structured 6-session Solution-Focused Brief Group Counseling program was designed specifically for adolescents. Consistent with similar evidence-based applications in the literature, each session was planned to last 90 min, held once a week.

Session Summaries:

- Session 1 (Welcome/Orienting): Creating group rules, introducing the concept of self-confidence, helping members determine individual goals, and drawing attention to positive change using the “Miracle Question” technique.
- Session 2 (Awareness and Solutions): Exploring pre-session changes, reframing negative self-perceptions, and using “Scaling Questions” to help clients visualize their progress and set manageable steps.
- Session 3 (Strengths and Boundaries): Highlighting the importance of saying “No.” Using “Exception Questions” to identify times when self-confidence was not an issue, and reinforcing positive steps with the “Compliment” technique.
- Session 4 (Assertiveness Training): Differentiating among avoidant, aggressive, and assertive behaviors. Practicing assertiveness to directly build self-confidence.
- Session 5 (Problem-Solving Skills): Equipping clients with problem-solving tools, encouraging the “Search for Solution” mindset, and rehearsing coping strategies for difficult situations.
- Session 6 (Termination): Assessing the achievement of initial goals using “Scaling Questions”, providing mutual positive feedback (“Love Bombing” activity), and finalizing the termination process.

### 2.5. Data Analysis

Prior to hypothesis testing, the data were examined to ensure that the assumptions for parametric tests were met. The Shapiro–Wilk test indicated that the self-confidence scores were normally distributed across all pre-test, post-test, and follow-up measurements for both the low and moderate self-confidence groups (*p* > 0.05 for all). Additionally, Levene’s test confirmed the homogeneity of variances among the groups across all time points (*p* > 0.05).

A 2 × 3 mixed design Analysis of Variance (Repeated Measures ANOVA) was conducted to evaluate the main and interaction effects of the intervention over time. Scheffe’s post hoc test was utilized to determine exactly where the significant differences occurred between measurements. All statistical analyses were performed using SPSS 17.0, with a significance level set at *p* < 0.05.

## 3. Results

This section presents the detailed statistical analyses conducted to test the hypotheses and the obtained findings.

### 3.1. Results Related to Adolescents with Low Self-Confidence

The first hypothesis of the study posited that “there will be a significant increase in the self-confidence levels of adolescents with low self-confidence in the experimental group compared to the control group, and this result will correspond to a lasting effect in the follow-up measurement.”

Before testing this hypothesis, the arithmetic means and standard deviations for the pre-test, post-test, and follow-up measurements were calculated for both the experimental and control groups with low self-confidence. The results are presented in Table 3.

As seen in Table 3, there is a clear increase in the post-test and follow-up test mean scores of adolescents in the experimental group compared to their pre-test scores. In contrast, the pre-test, post-test, and follow-up averages for the control group remained very close to each other, indicating no substantial change. To test the primary hypothesis, a Repeated Measures ANOVA was conducted to determine if the differences were statistically significant.

As shown in Table 4, the group effect was found to be significant (F(1, 22) = 59.872, *p* < 0.05). This indicates a significant difference between the experimental and control groups regardless of the time of measurement. Furthermore, the interaction effect of Intervention × Time, which is crucial for this study, was also significant (F(2, 44) = 59.732, *p* < 0.05). The partial eta squared value (η^2^ = 0.731) indicates a large effect size, suggesting that the intervention explains a substantial proportion of the variance in self-confidence scores.

To determine exactly where these differences occurred, Scheffe’s post hoc test was performed (Table 5).

Table 5 confirms the sub-hypothesis: there was a significant difference ($\bar{X}$ = −28.25, *p* < 0.05) between the pre-test ($\bar{X}$ = 79.75) and post-test ($\bar{X}$ = 108.00) scores of the experimental group. Similarly, the difference between pre-test and follow-up scores ($\bar{X}$ = 110.75) was significant ($\bar{X}$ = −31.00, *p* < 0.05). Crucially, no significant difference was found between the post-test and follow-up scores ($\bar{X}$ = −2.75, *p* > 0.05), indicating that the gains in self-confidence were maintained over time. The control group showed no significant changes.

**Figure 1:**
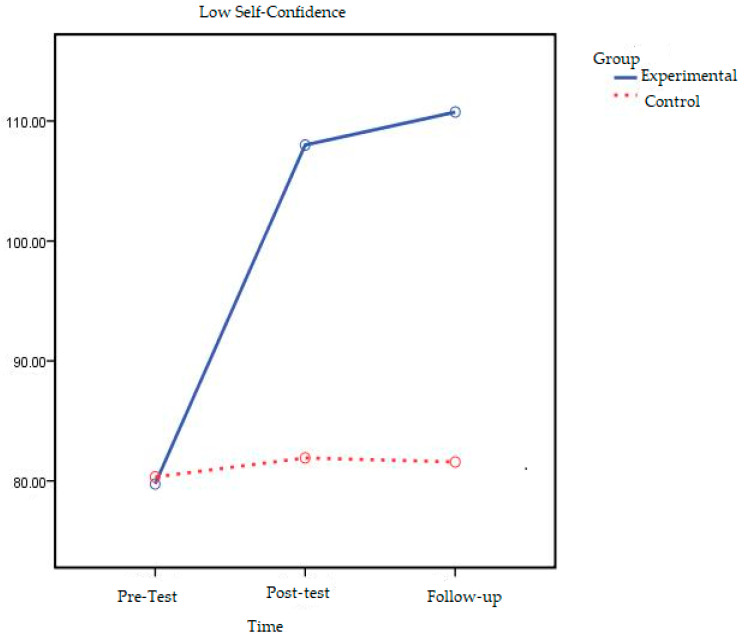
Graph of low self-confidence experimental and control groups’ pre-test, post-test, and follow-up test Self-Confidence Score averages.

The line chart of the change in pre-test, post-test, and follow-up test scores of low self-confidence experimental and control groups is shown in Figure 1.

When Figure 1 is examined, it can be seen that the self-confidence scores of adolescents in the low self-confidence experimental group changed after the experimental procedure and that this change continued 2 months after the experimental procedure was completed. On the other hand, it can be seen that there was no significant difference between the self-confidence pre-test, post-test, and follow-up test measurements of the adolescents in the control group and that the difference was horizontal.

When all the results obtained were assessed, it was found that the self-confidence levels of the low self-confidence experimental group adolescents who participated in short term solution-oriented group counseling changed and this change was found to continue in follow-up measurements conducted two months later. No significant change was found in the self-confidence levels of adolescents in the control group. For this reason, it can be said that the primary hypothesis of the study, which was “there will be a significant increase in self-confidence levels of adolescents who participated in Solution-Focused Brief Counseling when compared with the control group and this result will continue in follow-up measurement to be conducted two months later the practices are completed”, was supported.

### 3.2. Results Related to Adolescents with Moderate Self-Confidence

The second hypothesis stated that “there will be a significant increase in the self-confidence levels of adolescents with moderate self-confidence in the experimental group compared to the control group.” The descriptive statistics are presented in Table 6.

As can be understood from the results, the mean post-test scores of the adolescents in the experimental group with moderate self-confidence were significantly higher than the pre-test scores. Likewise, the mean scores of the follow-up test were significantly higher than the pre-test scores. It can be seen that the self-confidence pre-test, post-test, and follow-up test averages of adolescents in the moderate self-confidence control group are very close to each other; in other words, there is no change. To test the significance of these changes, a Repeated Measures ANOVA was conducted (Table 7).

As seen in Table 7, the Intervention × Time interaction effect was significant (F(2, 44) = 24.393, *p* < 0.05), with a large effect size (η^2^ = 0.526). This confirms that the change in self-confidence scores over time differed significantly depending on whether the participants received the intervention. In order to find out in which groups this difference occurred, Scheffe test values of adolescents in moderate self-confidence experimental and control groups obtained from self-confidence pre-test, post-test, and follow-up test between groups and between measurements are shown in Table 8.

According to Table 8, a significant increase was observed in the experimental group from pre-test ($\bar{X}$ = 98.00) to post-test ($\bar{X}$ = 115.50) (*p* < 0.05), and this increase was maintained at follow-up ($\bar{X}$ = 115.25). No statistically significant change occurred between the post-test and follow-up measurements ($\bar{X}$ = 0.25, *p* > 0.05), confirming the permanence of the effect.

**Figure 2:**
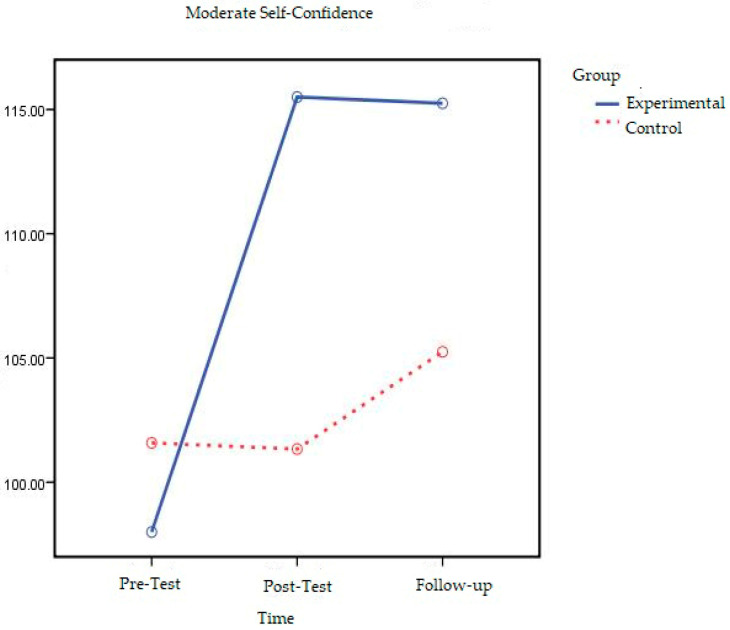
Graph of moderate self-confidence experimental and control groups’ pre-test, post-test, and follow-up test Self-Confidence Score averages.

The line chart of the change in the pre-test, post-test, and follow-up test scores of moderate self-confidence experimental and control groups is shown in Figure 2.

When Figure 2 is examined, it can be seen that the self-confidence scores of adolescents in the moderate self-confidence experimental group changed after the experimental procedure and that this change continued 2 months after the experimental procedure was completed. On the other hand, it can be seen that there was no significant difference between the self-confidence pre-test, post-test, and follow-up test measurements of the adolescents in the control group and that the difference was horizontal.

When all the results obtained were assessed, it was found that the self-confidence levels of the moderate self-confidence experimental group adolescents who participated in short-term solution-oriented group counseling changed and this change was found to continue in follow-up measurements conducted two months later. No significant change was found in the self-confidence levels of adolescents in the control group. For this reason, it can be said that the primary hypothesis of the study, which was “there will be a significant increase in self-confidence levels of adolescents who participated in Solution-Focused Brief Counseling when compared with the control group and this result will continue in follow-up measurement to be conducted two months later the practices are completed”, was supported.

## 4. Discussion and Conclusions

### 4.1. Discussion of the Findings

The primary hypothesis of the study stated that adolescents participating in Solution-Focused Brief Counseling would demonstrate a significant increase in their self-confidence levels compared to the control group, and these gains would be maintained during the follow-up period. The findings suggest that the intervention was associated with significant improvements under these conditions. Post-test self-confidence scores for both the low and moderate self-confidence experimental groups were significantly higher than their pre-test scores. Furthermore, this increase was maintained during the follow-up measurements, suggesting that the intervention facilitated a lasting positive effect on the participants’ self-confidence.

While the previous literature extensively highlights the general efficacy of the solution-focused approach across various domains, such as decreasing burnout (İlbay, 2014), reducing peer bullying (Çitemel, 2014), and preventing risk-taking behaviors (Uysal, 2014), this study offers a unique contribution. What makes this study unique is its direct and isolated focus on “self-confidence” as a primary variable, tested simultaneously across different baseline severities (low vs. moderate) within a randomized controlled group design.

### 4.2. Mechanisms of Change, Group Dynamics, and Developmental Factors

To understand why SFBT enhances self-confidence, it is crucial to unpack the mechanisms of change, group dynamics, and developmental factors in adolescents. Adolescents go through an active developmental period where they often resist deeply discussing their problems, perceiving such analysis as criticism from the adult world (Meydan, 2013). SFBT naturally aligns with their developmental need for autonomy and competence. By bypassing the “problem-talk” and directly engaging in “solution-talk”, the intervention validates their current strengths.

The specific mechanisms of SFBT play a direct role in this cognitive shift. For instance, the “miracle question” technique shifts the adolescent’s focus from current deficits to future possibilities, fostering a sense of hope. “Scaling questions” break down overwhelming goals into manageable steps, creating repeated experiences of small successes that directly build self-efficacy. Furthermore, applying these mechanisms within a group dynamic amplified the therapeutic effects. Adolescence is a period where peer validation is paramount. Being in a group allowed members to witness their peers’ successes, normalizing their own struggles. Techniques such as ‘Love Bombing’ and mutual compliments utilized positive peer influence, providing external validation that internalized into higher self-confidence.

These results are consistent with the broader literature. La Fountain and Garner found that solution-oriented programs applied by school counselors helped students develop more positive attitudes toward themselves (Uysal, 2014). Similarly, Springer et al. (2000) and Leggett (2004) reported significant developments in students’ self-esteem following solution-focused interventions. Given that self-esteem and self-confidence are closely related self-constructs, these findings strongly support the current study’s outcomes. Furthermore, studies by Newsome (2005) and Kvarme et al. (2010) demonstrated that SFBT enhances social skills and self-sufficiency, which are behavioral manifestations of increased self-confidence.

### 4.3. Limitations and Future Directions

While the findings are promising, several methodological limitations must be acknowledged to provide a balanced discussion.

- Sample Size and Sampling: The study utilized a relatively small sample size (n = 48 total; n = 12 per subgroup). Additionally, the use of single-site sampling (one high school in a specific province) and its cultural specificity to the Turkish educational context limit the generalizability of the findings to broader or more diverse populations.
- Lack of an Active Control Group: The control groups in this study received no intervention. Without an active control group (e.g., a group receiving an alternative psycho-educational activity or general counseling), it is difficult to completely rule out whether the improvements were due to the specific SFBT techniques or simply the result of receiving attention and being part of a group (the Hawthorne effect).
- Researcher Effects: The intervention was administered by the researcher, which introduces the potential for researcher expectancy effects. The researcher’s enthusiasm and belief in the therapy might have influenced the participants’ responses.

Future research should address these limitations by utilizing larger, multi-site samples across different cultural contexts. Employing an active placebo control group and utilizing multiple independent therapists blind to the study’s hypotheses would significantly strengthen the causal claims regarding SFBT’s efficacy. Additionally, while self-report scales are valuable, future studies could incorporate multidimensional measurements, including teacher or parent observations, to capture a more holistic view of adolescents’ self-confidence.

### 4.4. Conclusions

In conclusion, the findings suggest that Solution-Focused Brief Group Counseling is a practical and developmentally appropriate approach associated with meaningful improvements in the self-confidence levels of adolescents. Because it is brief, cost-effective, and focuses on strengths rather than pathologies, school counselors can effectively integrate this program into their regular counseling plans starting from middle school to support students’ psychological well-being.

## Figures and Tables

**Table 1:** Experimental study design.

Groups	Pre-Test	Procedure	Post-Test	Follow-Up
Experimental groups	SS *	Solution-Focused Brief Counseling Application (6 sessions)	SS	SS
Control groups	SS	No procedure	SS	SS

* SS: Self-confidence scale.

**Table 2:** Distribution of individuals in experimental and control groups in terms of gender and grade.

		Low Self-Confidence		Moderate Self-Confidence	
		Experimental	Control	Experimental	Control
Gender	Female	5	4	6	6
	Male	7	8	6	6
Grade	9	3	4	2	7
	10	4	4	7	2
	11	5	4	3	3

**Table 3:** Means and standard deviations of Self-Confidence Scores for low self-confidence groups.

Group	Pre-Test			Post-Test			Follow-Up Test		
	N	$\bar{X}$	S	N	$\bar{X}$	S	N	$\bar{X}$	S
Experimental	12	79.75	2.34	12	108	11.92	12	110.75	9.09
Control	12	80.33	1.66	12	81.91	5.99	12	81.58	6.17

**Table 4:** Repeated Measurements ANOVA results of low self-confidence experimental and control groups.

Source of the Variance	Sum of Squares	Sd	Average of Squares	F	*p*	η^2^
Between groups	8173.111	23				
Intervention (Experimental/Control)	5976.889	1	5976.889	59.872	0.000	0.731
Error	2196.222	22	99.828			
Intragroup	8266	48				
Time (pre-test, post-test, follow-up test)	3871.861	2	1935.931	72.018	0.000	0.766
Intervention × Time	3211.361	2	1605.681	59.732	0.000	0.731
Error	1182.778	44	26.881			

**Table 5:** Scheffe test results of between groups and between measurements of Self-Confidence Scale Scores in adolescents with low self-confidence.

		Experimental Group			Control Group		
		Pre-Test	Post-Test	Follow-Up	Pre-Test	Post-Test	Follow-Up
Experimental	Pre-Test	-	−28.25 **	−31.00 **			
	Post-Test		-	−2.75		26.09 **	
	Follow-up			-			29.17 **
Control	Pre-Test				-	−1.58	−1.25
	Post-Test					-	0.33
	Follow-up						-

** *p* < 0.05.

**Table 6:** Means and standard deviations of Self-Confidence Scores for moderate self-confidence groups.

Group	Pre-Test			Post-Test			Follow-Up Test		
	N	$\bar{X}$	S	N	$\bar{X}$	S	N	$\bar{X}$	S
Experimental	12	98	7.45	12	115.50	11.40	12	115.25	10.50
Control	12	101.58	5.93	12	101.33	7.02	12	105.25	7.16

**Table 7:** Repeated Measurements ANOVA results of moderate self-confidence experimental and control groups.

Source of the Variance	Sum of Squares	Sd	Average of Squares	F	*p*	η^2^
Between groups	4665.986	23				
Intervention (Experimental/Control)	847.347	1	847.347	4.882	0.038	0.182
Error	3818.639	22	173.574			
Intragroup	3463.333	48				
Time (pre-test, post-test, follow-up test)	1497.028	2	748.514	35.321	0.000	0.616
Intervention × Time	1033.861	2	516.931	24.393	0.000	0.526
Error	932.444	44	21.192			

**Table 8:** Scheffe test results of between groups and between measurements of Self-Confidence Scale scores in adolescents with moderate self-confidence.

		Experimental Group			Control Group		
		Pre-Test	Post-Test	Follow-Up	Pre-Test	Post-Test	Follow Up
Experimental	Pre-Test	-	−17.50 **	−17.25 **			
	Post-Test		-	0.25		14.17 **	
	Follow up			-			10.00
Control	Pre-Test				-	0.25	−3.67
	Post-Test					-	−3.92
	Follow up						-

** *p* < 0.05.

## Data Availability

The data presented in this study are available on request from the corresponding author. The data are not publicly available due to privacy restrictions.
